# Infliximab in neurosarcoidosis: a systematic review and meta‐analysis

**DOI:** 10.1002/acn3.51968

**Published:** 2023-12-12

**Authors:** Siwakorn Chaiyanarm, Piraya Satiraphan, Natnasak Apiraksattaykul, Jiraporn Jitprapaikulsan, Weerapat Owattanapanich, Tarinee Rungjirajittranon, Witsarut Nanthasi

**Affiliations:** ^1^ Faculty of Medicine Siriraj Hospital Mahidol University Bangkok 10700 Thailand; ^2^ Siriraj Neuroimmunology Center, Division of Neurology, Department of Medicine, Faculty of Medicine Siriraj Hospital Mahidol University Bangkok 10700 Thailand; ^3^ Division of Neurology, Department of Medicine, Faculty of Medicine Siriraj Hospital Mahidol University Bangkok 10700 Thailand; ^4^ Division of Hematology, Department of Medicine, Faculty of Medicine Siriraj Hospital Mahidol University Bangkok 10700 Thailand

## Abstract

**Objectives:**

To evaluate the clinical outcomes and relapse rates in neurosarcoidosis patients administered infliximab.

**Methods:**

A systematic review was conducted using the MEDLINE, EMBASE, SCOPUS, and Cochrane Library databases. The search included studies from their inception to March 2023. We included case‐series studies with at least 10 neurosarcoidosis patients undergoing any treatment type. Studies were also required to report at least one of the following outcomes: response rate, overall survival rate, or relapse rate. This study adhered to the Preferred Reporting Items for Systematic Reviews and Meta‐Analyses guidelines. A random‐effects model facilitated the analysis of proportional treatment outcomes. Study quality was evaluated using the modified Newcastle–Ottawa quality assessment scale, while a funnel plot helped detect any publication bias.

**Results:**

Seven studies comprising 237 patients with neurosarcoidosis were included in the analysis. Of these patients, 184 (77.6%) received treatment with infliximab. The pooled proportion of patients showing clinical improvement after infliximab treatment was 0.74 (95% CI 0.64–0.84, *I* ^2^ = 49.73%). Relapse rates, derived from four studies, stood at 0.38 (95% CI 0.22–0.55, *I* ^2^ = 56.92%). Most studies reported successful tapering or cessation of corticosteroid dosage in patients receiving infliximab. Adverse effects were reported in 52 (29.4%) patients, of which 39 out of 54 events (72.2%) were linked to infections.

**Interpretation:**

Infliximab demonstrated potential improvement in clinical outcomes for patients with refractory neurosarcoidosis and showed potential for reducing the dosage of concurrent corticosteroids. However, a degree of relapse was observed, with infections being the primary concern for adverse events.

## Introduction

Sarcoidosis is a multisystemic granulomatous disease of unknown etiology that can affect various organ systems.^1^ Between 5% and 10% of systemic sarcoidosis cases involve the neurological system,^2^ leading to a disproportionate amount of disability and an unfavorable prognosis.^3^

Traditionally, the treatment of sarcoidosis commences with corticosteroids as the first‐line approach. However, the evidence supporting the efficacy of this treatment remains inconsistent. When patients experience partial responses or require long‐term steroid therapy, second‐line immunosuppressive agents such as hydroxychloroquine, azathioprine, cyclophosphamide, or methotrexate are often prescribed.^4^ Recent research has highlighted the potential of tumor necrosis factor‐alpha (TNF‐α) inhibitors in managing refractory neurosarcoidosis.^5^

TNF‐α is a central proinflammatory cytokine instrumental in the formation and sustenance of granulomas.^6^ Infliximab, a chimeric mouse‐human monoclonal antibody targeting TNF‐α, has shown some effectiveness in treating sarcoidosis, although the available studies have not provided strong evidence to support a high degree of confidence in its efficacy. It has been employed specifically to address cases of steroid‐dependent or refractory neurosarcoidosis. Infliximab therapy aims to achieve remission and is typically extended over several years for patients with severe sarcoidosis manifestations.^7^

We conducted a systematic review and meta‐analysis to assess the clinical outcomes and relapse rate of neurosarcoidosis patients treated with infliximab.

## Materials and Methods

### Search strategy

The review protocol was registered with PROSPERO, the International Prospective Register of Systematic Reviews (https://www.crd.york.ac.uk/prospero/, identifier: CRD42022300320). Two independent researchers (S.C., P.S.) performed a comprehensive search of the MEDLINE, EMBASE, SCOPUS, and Cochrane Library databases from their inception to March 2023. The search terms were related to “neurosarcoidosis” and treatments, including “infliximab.” All search terms used can be found in the Supplementary Data. The study adhered to the Preferred Reporting Items for Systematic Reviews and Meta‐Analyses (PRISMA) guidelines.^8^

### Study identification and selection

Two researchers (S.C., P.S.) initially screened the studies based on their titles and abstracts, with a third investigator (N.A. or W.N.) resolving any conflicts during the screening process. Covidence software was utilized for data screening and extraction. Studies were considered for initial screening based on their title and abstract if they:

- Diagnosed neurosarcoidosis according to the Zajicek or 2018 NCGG criteria.
- Were retrospective or prospective studies (*n* ≥ 10).
- Included neurosarcoidosis patients undergoing any form of treatment.
- Administered pharmacological treatments that spanned first‐line, second‐line, and third‐line therapies.
- Reported at least one of these outcomes: response rate, overall survival rate, or relapse rate.
- Addressed sarcoidosis patients with neurological manifestations not attributable to other diseases.

Studies were excluded if they:

- Were deemed irrelevant.
- Were categorized as news, letters, books, documentation, reviews, meta‐analyses, or were not peer‐reviewed articles.
- Were published in languages other than English.
- Did not provide clear reports on treatment outcomes with infliximab.

After the initial title and abstract screening, the same investigators conducted a full review of the included studies, applying further exclusion criteria:

- The treatment's efficacy could not be evaluated.
- The outcomes were ambiguously reported.
- Neurological manifestations in sarcoidosis patients were more fittingly attributed to other conditions.
- The evidence substantiating the diagnosis was dubious.

The final number of studies included in the meta‐analysis was determined after the full review.

### Data extraction

Two researchers (S.C., P.S.) extracted the following data:

- Study design.
- Sample characteristics, including sex distribution, total number of patients, age at onset or initiation of infliximab treatment, and diagnostic criteria.
- Treatment specifics, including the number of patients treated with infliximab, dosage, and accompanying immunosuppressive medications.
- Treatment outcomes, specifically, the rate of improvement and relapse. Clinical improvement was defined as partial response or remission.
- Adverse events observed in patients treated with infliximab.

### Quality assessment and statistical analysis

We employed the modified Newcastle–Ottawa quality assessment scale to gauge the quality of the included observational studies. This scale consists of two domains: the selection of participants and the ascertainment of outcomes in cohort studies. Since all the studies were either observational or one‐arm, the comparability domain was not applicable. Any discrepancies in the assessment were resolved through consensus among the investigators. A funnel plot was employed to visualize any potential publication bias in the included studies.

The primary outcome under consideration was the overall clinical improvement and clinical remission observed in patients administered infliximab. For analysis, data from different studies were pooled and reported 95% confidence intervals (CIs) using a random‐effects model. All statistical evaluations were executed using STATA/MP 14.0 (Stata Corp, College Station, TX, USA). Forest plots provided a visual representation of the study's results. The *I* ^2^ index was used to gauge the heterogeneity across studies. *I* ^2^ values of 0%–25%, 26%–75%, and 75%–100% correspond to low, moderate, and high levels of heterogeneity, respectively.^9^ Due to the low number of studies included in the meta‐analysis, meta‐regression or sensitivity analyses were not performed.

## Results

**Figure 1:**
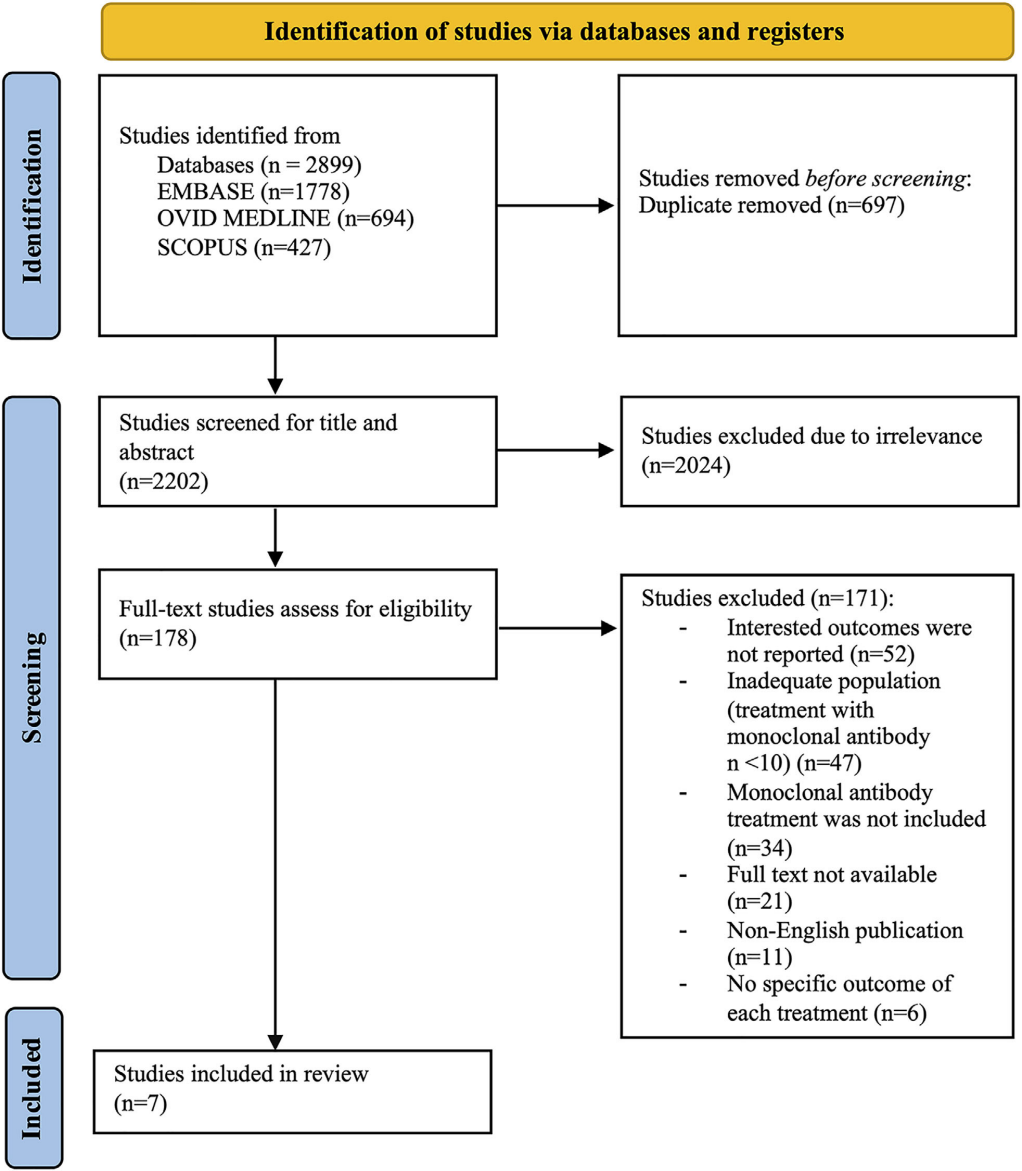
Flowchart detailing the systematic review process. This figure demonstrated the flowchart detailing the systematic review process. After the identification, 2202 studies were included to screen for title and abstract. Then, 178 studies were eligible for full‐text assessment. In the conclusion, 7 studies were included in the review process.

An initial retrieval yielded 2899 studies from the MEDLINE, EMBASE, SCOPUS, and Cochrane Library databases. After removing 697 duplicate articles and 2024 irrelevant articles, 178 articles underwent full‐text reviews. Applying the exclusion criteria eliminated another 171 articles (Fig. 1). This meant that seven retrospective studies met the criteria for inclusion in the analysis.

Quality assessment of the remaining seven studies was conducted using the modified Newcastle–Ottawa scale. Two studies scored 7 out of 9 stars, while five received 6 out of 9 stars. Due to the exclusive inclusion of observational studies, the comparability aspect could not be assessed (eTable 1 in the Supplement).

Standard errors and effect sizes from the selected studies were plotted on funnel plots to evaluate the possibility of publication bias. The plots showed a symmetrical distribution, indicating the absence of significant publication bias. Detailed figures are provided in the Supplementary Data (Figs. S1 and S2).

From the seven studies analyzed, 237 patients were diagnosed with neurosarcoidosis. The ages of the patients ranged from 20 to 77 years. Slightly over half of the patients were female (56.5%, [134/237]). Table 1 summarizes the demographic data and characteristics of the studies in terms of age, sex ratio, diagnostic criteria, treatment details, and treatment outcomes.

**Table 1:** Characteristics of the seven studies involving patients diagnosed with neurosarcoidosis who were treated with infliximab.

References	Study design	Total no. of patients	Number of female/total number (%)	Age, years old	Diagnostic criteria	Number of infliximab treatment (%)	Dosage of infliximab, number of patients (%)	Other immunosuppressive treatments, number of patient (%)	Clinical outcomes in patients received infliximab, number of patients (%)	Relapse rate in infliximab, n of patients (%)
Chakales et al. (2022)^10^	Retrospective	26	10/26 (38.5%)	Median 43.5 (range 22.0–70.0)	2018 NCCG	7 (26.9%)	N/A	MTX, 12 (46.1%) MMF, 7 (26.9%) AZA, 6 (23.1%) RTX, 5 (19.2%) ADA, 1 (3.8%) CYP, 1 (3.8%)	Improved, 6/7 (85.7%) Failed, 1/7 (14.3%)	N/A
Hilezian et al. (2021)^11^	Retrospective	23	13/23 (56.5%)	Mean 41.5 (SD 10.5)	2018 NCCG	22 (95.6%)	5–10 mg/kg every 4–10 weeks (most common 8 mg/kg every 8 weeks)	CS, 23 (100%) MTX, 8 (34.8%) CYP, 4 (17.4%) AZA, 2 (8.7%) ADA, 1 (4.3%)	(All TNF‐alpha inhibitor) Improved (61%) Stable (30%) Worsening, 2/23 (9%)	N/A
Fritz et al. (2020)^5^	Retrospective	28	12/28 (42.8%)	Mean 43.0 (SD 11.6)	Zajicek criteria	28 (100%)	5 mg/kg every 4 weeks, 5 (18%) 5 mg/kg every 6 weeks, 12 (43%) 5 mg/kg every 8 weeks, 11 (39%)	CS, 11 (39.3%) CS+MTX, 6 (21.4%) CS+AZA, 4 (14.3%) CS+MMF+MTX, 2 (7.1%) CS+HCQ 1 (3.6%) None 3 (10.7%), receive immunosuppressive medication previously	Complete remission, 6/28 (21%) Improved, 14/28 (50%) Stable, 7/28 (25%) Deteriorated, 1/28 (4%)	6/28 (21.4%) (5 stopped or decreased dose)
Lord et al. (2020)^12^	Retrospective	56	35/56 (62.5%)	Mean 49.0 (range 22.0–77.0)	2018 NCCG	23 (41.1%)	N/A	CS, 51 (91.1%) MTX, 26 (46.4%) AZA, 13 (23.2%) MMF, 6 (10.7%) RTX, 4 (7.1%)	Improved, 11/23 (47.8%) Stable, 9/23 (39.1%) Failed, 3/23 (13.0%)	N/A
Riller et al. (2019)^13^	Retrospective	20	9/20 (45%)	Median 37.0 (IQR 31.0–45.0)	2018 NCCG	20 (100%)	5 mg/kg with intervals 2 and 4 weeks (initiation group) 5 mg/kg with intervals vary between 4 and 8 weeks (switching group)	CS+MTX, 12 (60%) CS, 4 (25%) CS+AZA, 2 (10%) HCQ+MTX, 1 (5%) MTX, 1 (5%)	First 6 months (*n* = 20) Remission, 17/20 (85%) Relapse/progress, 3/20 (15%) After 6 months (*n* = 17, biosimilar) Remission, 14/17 (82.3%) Relapse/progress, 3/17 (17.6%)	6/20 (30%)
Cohen Aubart et al. (2017)^14^	Retrospective	18	7/18 (38.9%)	Median 38.0 (Range 27.0–51.0)	Zajicek criteria	18 (100%)	3 mg/kg, 1 (5.5%) 5 mg/kg, 16 (88.9%) 7.5 mg/kg, 1 (5.5%) Interval of 2 weeks	MTX+CS, 15 (83.3%) AZA+CS, 2 (11.1%) MMF+CS, 1 (5.5%)	Complete remission, 6/18 (33.3%) Partial remission, 10/18 (55.5%) Stable, 2/18 (11.1%)	9/18 (50%) (4 after infliximab withdrawal)
Gelfand et al. (2017)^15^	Retrospective	66	48/66 (72.7%)	Mean 47.5 (SD 11.7)	Zajicek criteria	66 (100%)	Varied loading dose. Maintenance dose ranged from 3 to 7 mg/kg and frequency ranged from every 4 to 8 weeks	CS, 52 (80%) MTX, 22 (33.3%) MMF, 18 (27.3%) AZA, 10 (15.1%) Others, 2 (3.0%)	Complete recovery, 19/66 (28.8%) Improved, 32/66 (48.5%) Stable, 12/66 (18.2%) Worsening, 2/66 (3%) Loss to follow‐up, 1/66 (1.5%)	9/16 (56.2%) (Discontinued infliximab after remission)

2018 NCCG, 2018 Neurosarcoidosis Consortium Consensus Group; ADA, adalimumab; AZA, azathioprine; CS, corticosteroids; CYP, cyclophosphamide; HCQ, hydroxychloroquine; MMF, mycophenolate mofetil; MTX, methotrexate; N/A, not available; RTX, rituximab.

**Figure 2:**
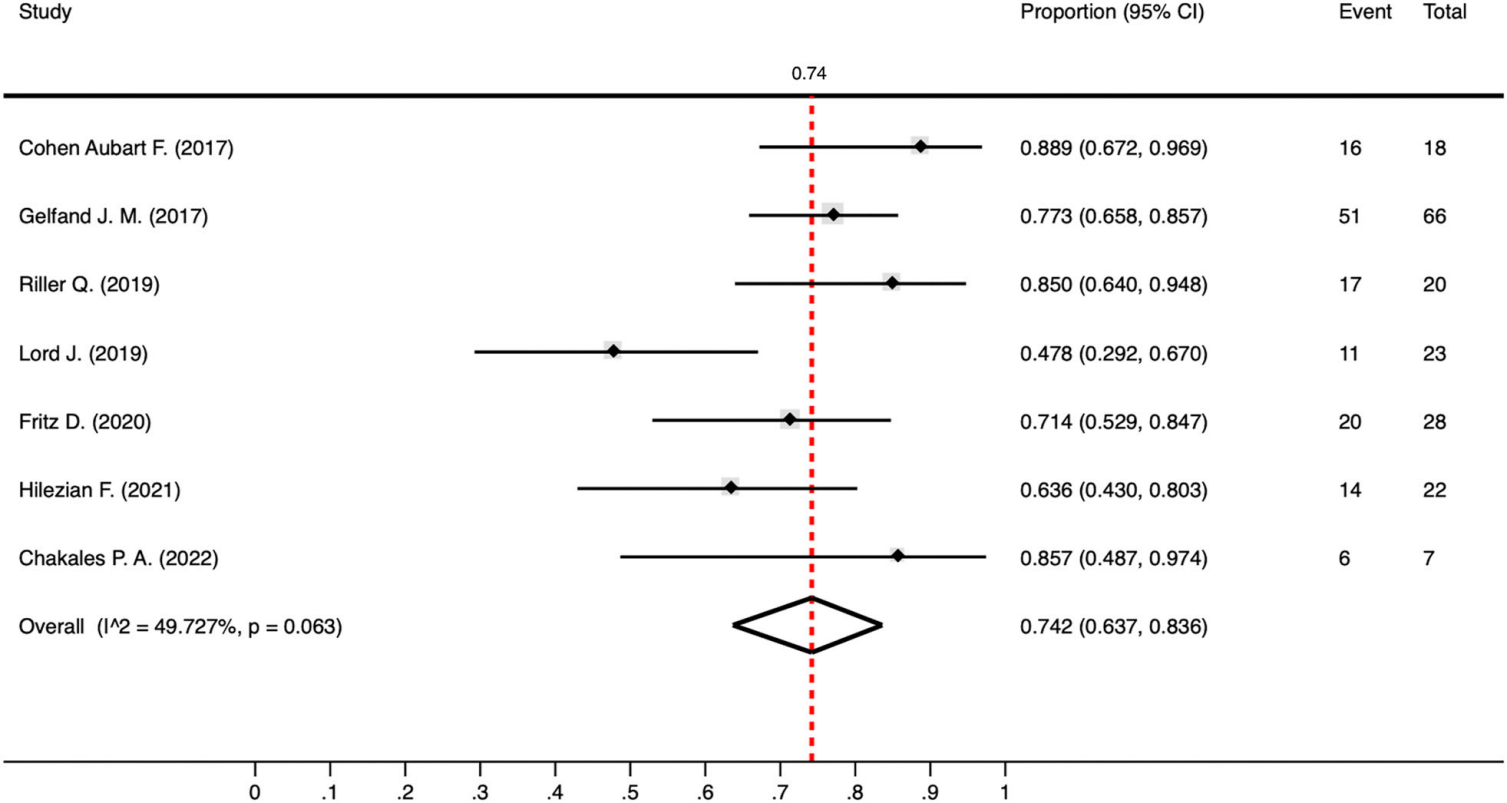
Forest plot showing the proportion of clinical improvement among neurosarcoidosis patients treated with infliximab. This figure demonstrated the proportion of clinical improvement in neurosarcoidosis patients treated with infliximab. The pooled proportion was 0.74 (95% CI 0.64–0.84, *I* ^2^ = 49.73%).

**Figure 3:**
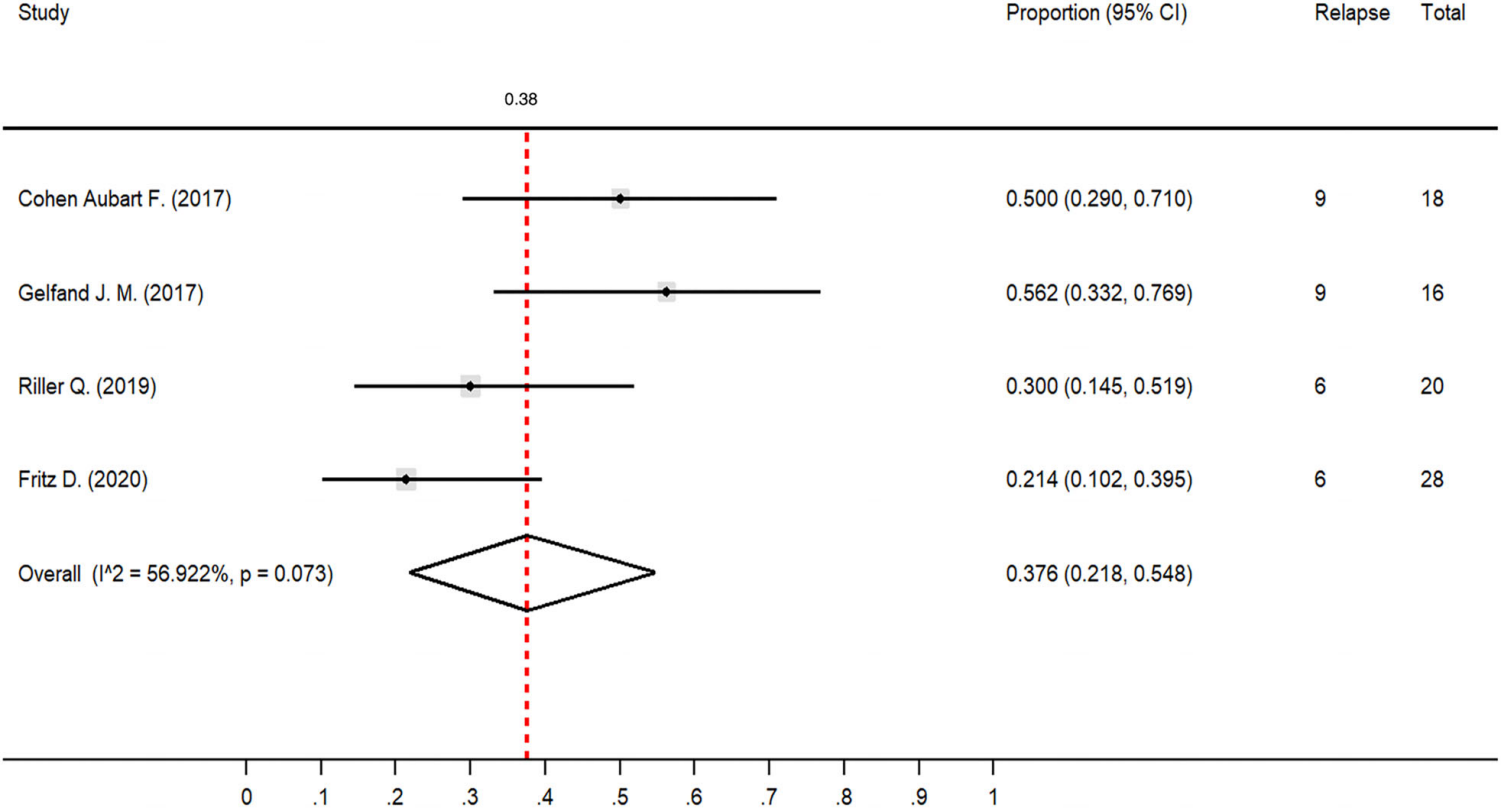
Forest plot showing the proportion of clinical relapse rate among neurosarcoidosis patients treated with infliximab. This figure demonstrated the proportion of clinical relapse in neurosarcoidosis patients treated with infliximab. The pooled proportion was 0.38 (95% CI 0.22–0.55, *I* ^2^ = 56.92%). This result came from 4 mentioned studies.

Using infliximab resulted in noticeable improvements in clinical outcomes and relapse rates for neurosarcoidosis patients. Of the 237 patients, 184 (77.6%) were administered infliximab. The pooled proportion of patients who exhibited improved clinical outcomes after receiving infliximab was 0.74 (95% CI 0.64–0.84, *I* ^2^ = 49.73%; Fig. 2). Additionally, a relapse rate of 0.38 (95% CI 0.22–0.55, *I* ^2^ = 56.92%; Fig. 3) was calculated based on data from four of these studies: Cohen Aubart et al.,^14^ Gelfand et al.,^15^ Riller et al.,^13^ and Fritz et al.^5^ Upon the reduction or discontinuation of infliximab, 18 out of 30 patients (60%) experienced a relapse. Conversely, 12 patients reported clinical relapses while still undergoing infliximab therapy.

The infliximab dosage and concurrent use of other immunosuppressive medications in the included studies differed. Infliximab doses ranged from 3 to 10 mg/kg and administered at intervals spanning 2 to 10 weeks. Detailed information on medication dosages in each study is shown in Table 1.

Most studies reported tapering or discontinuation of corticosteroids following infliximab initiation. Five studies provided data on changes in corticosteroid dosage after the start of infliximab treatment (Table 2). The majority of these studies reported that patients reduced their corticosteroid intake to a median of 5 mg/day. Cohen Aubart et al.^14^ and Gelfand et al.^15^ documented 89% and 27% success rates, respectively. Hilezian et al.^11^ reported that 65% of patients managed to reduce their corticosteroid dose to under 6 mg/day. Hilezian et al.^11^ and Gelfand et al.^15^ mentioned the complete discontinuation of corticosteroids in 23% and 40% of their patient populations using the medication, respectively. Fritz et al. combined their data on dose tapering and discontinuation, indicating a combined rate of 68%. Riller et al., however, did not specify a proportion.

**Table 2:** Adjustments in corticosteroid doses in five studies examining infliximab treatment for neurosarcoidosis patients.

References	Number of infliximab treatment	Number of concurrent corticosteroid usage (%)	Corticosteroid dose adjustment after infliximab initiation	Number of patients with corticosteroid dose tapering (%)
Chakales et al. (2022)^10^	7	N/A	N/A	N/A
Hilezian et al. (2021)^11^ ^,^ ^a^	22	23 (100)	<9 mg/day	18/23 (78.3)
			<6 mg/day	15/23 (65.2)
			Cessation	6/23 (26.1)
Fritz et al. (2020)^5^	28	24 (85.7)	Tapering or discontinuation without relapse	19/24 (79.2)
Riller et al. (2019)^13^	20	18 (90)	Reduced median steroid usage from daily dose of 11.5 mg at baseline to 5 mg at the time of last follow‐up	N/A
Lord et al. (2020)^12^	23	N/A	N/A	N/A
Cohen Aubart et al. (2017)^14^	18	18 (100)	5 mg/day (at last visit)	16/18 (88.9)
Gelfand et al. (2017)^15^	66	52 (78.8)	5 mg/day or less	18/52 (34.6)
			Discontinue	27/52 (51.9)

N/A, not available.

^a^ Reported as all TNF‐alpha inhibitors, including 22 treated with infliximab and 1 with adalimumab.

Infections were the most cited adverse event. Of the six studies that reported on a collective 177 patients, 52 patients (29.4%) experienced a total of 54 adverse events. A significant portion of the events (39/54, 72.2%) were related to infections. The reported sites of infection were the respiratory, urinary, and musculoskeletal systems. Other observed adverse reactions included allergic responses, liver test abnormalities, diarrhea, severe alopecia, and myositis. The range of treatment discontinuation rates due to these side effects ranged between 0% and 13% (Table 3).

**Table 3:** Adverse events reported in six studies examining infliximab treatment in neurosarcoidosis patients.

References	Number of infliximab treatment	Number of patients with adverse events (%)	Adverse events	Number of events (%)	Number of discontinuations due to adverse event (%)
Chakales et al. (2022)^10^	7	N/A			
Hilezian et al. (2021)^11^ ^,^ ^a^	22	10	Infection	10	3 (13%)
Fritz et al. (2020)^5^	28	10 (35.7%)	Infection	8	0 (0%)
			Elevated liver test	1	
			Allergic reaction	1	
Lord et al (2020)^12^	23	6 (26.1%)	Infusion reactions	4	N/A
			Infection (osteomyelitis, septic joint)	2	
Riller et al. (2019)^13^	20	10 (50%)	Infection	5	2 (10%)
			Infusion reaction (urticaria 4, headache 1, anaphylaxis 1)	6	
			Permanent diarrhea	1	
Cohen Aubart et al. (2017)^14^	18	8 (44.4%)	Infection	7	1 (5.5%)
			Severe alopecia	1	
Gelfand et al. (2017)^15^	66	8 (12.1%)	Infection	7	1 (1.5%)
			Myositis	1	

N/A, not available.

^a^ Reported as all TNF‐alpha inhibitors, comprising 22 infliximab treatments and 1 adalimumab treatment.

## Discussion

This study highlights the benefits of adding infliximab to the treatment regimen for patients with refractory neurosarcoidosis or those unresponsive to first‐line treatments such as corticosteroids or second‐line therapies like methotrexate, mycophenolate mofetil, and azathioprine. Specifically, 74.2% of patients treated with infliximab experienced improved clinical outcomes.

A systematic review and meta‐analysis from 2016 that focused on the clinical features, treatments, and outcomes of neurosarcoidosis patients did not report the proportional rate of positive outcomes in patients receiving TNF‐alpha antagonist therapy. The recommendation for using agents such as infliximab stemmed from a prior phase II RCT in patients with pulmonary sarcoidosis. In that study, 59% of the participants showed improved outcomes.^16^ In comparison, our investigation, which concentrated on infliximab therapy, presented a more substantial 74.2% clinical response rate. This elevated response suggests that infliximab may provide an additional benefit in improving outcomes for patients with neurosarcoidosis. Additionally, another study posited that coupling infliximab with other drugs could lead to maximum improvements on the modified Rankin Scale compared to other treatment approaches.^17^

The relapse rate among patients with neurosarcoidosis treated with infliximab showed statistically nonsignificant results with moderate heterogeneity. This finding indicates that moderate‐level effects may result from multiple unaccounted variables. Differences in treatment regimens, such as dose tapering, cessation after adverse events, or length of follow‐up, could contribute to this variability. One important factor to consider concerning the relapse rate is the cessation of infliximab after patients have achieved favorable outcomes. From the available data, 60% of relapses occurred due to infliximab discontinuation. This phenomenon was primarily observed in patients who initially showed favorable responses, although some studies did not report unfavorable cases as the cause of relapse. This incidence provides some insight, as mentioned in one of the included studies.

The prescribed dosages of infliximab varied, often based on individual clinical considerations. While a recommended dosage has been proposed for pulmonary sarcoidosis, the ideal dosage for neurosarcoidosis remains undefined. Without a standardized protocol, the decision regarding the infliximab dosage largely relies on clinical judgment.

Interestingly, corticosteroid dosages may be able to be reduced in patients initiated on infliximab therapy. For patients with less severe presentations or those receiving treatment to maintain treatment response, the suggested dose for oral prednisolone or its equivalent is 0.5–1 mg/kg/day or up to 40 mg/day.^4^, ^18^, ^19^ However, in patients with suboptimal responses, doses can escalate to as much as 100 mg/day,^19^ leading to potential systemic adverse effects such as Cushing syndrome and osteoporosis.^4^ Our analysis hints at the feasibility of reducing the corticosteroid dose to below 5 mg/day or even complete discontinuation among those treated with infliximab. Over half of the reviewed studies reported this possibility. While most relapses were attributed to stopping infliximab rather than tapering corticosteroid dosages, clinicians should still consider individual patient histories and profiles when deciding treatment strategies.

Previous studies have demonstrated the superiority of TNF‐alpha antagonists in treating the refractory state of the disease compared to other treatment modalities.^20^ This can be attributed to the role of TNF‐alpha in the pathogenesis of the disease, as it plays a crucial role in the interaction between T‐helper cells and macrophages, leading to the secretion of multiple interleukins and TNF‐alpha as mediators. This process ultimately contributes to the formation and maintenance of noncaseating granulomas, which are a histopathological characteristic of the disease.^21^ While other interleukins have also been implicated, such as IL‐1, IL‐2, IL‐12, IL‐15, and IL‐18,^21^, ^22^ TNF‐alpha remains prominent and has been consistently cited in multiple studies.

Infliximab, a representative therapy from the TNF‐alpha antagonist group, has been used to treat refractory patients who have not responded to corticosteroid or noncorticosteroid therapies.^20^ TNF‐alpha antagonists and other treatments aim to suppress the immune system, but they do so via distinct mechanisms. For instance, cyclosporin inhibits the amplification of the T‐helper cell immune response,^23^ whereas methotrexate directly affects T‐helper cells. Corticosteroids exhibit a broad spectrum of immunosuppressive actions; however, their potency in combatting aggressive diseases seems relatively subdued compared to other therapies.^24^ The superiority of TNF‐alpha antagonists may indicate the essential role of TNF‐alpha in the disease course, and our study's results support this hypothesis. However, the exact mechanism and molecular interactions between the disease and various treatments remain unclear and require further investigation.

The current treatment protocol for neurosarcoidosis typically involves corticosteroids as first‐line therapy and corticosteroid‐sparing immunosuppressive agents such as cyclosporin, mycophenolate mofetil, and methotrexate as second‐line options. TNF‐alpha antagonists, including infliximab, are considered third‐line treatments for patients with refractory disease. However, the existing treatment protocol has limitations. Conducting subgroup analyses based on different clinical presentations could potentially improve precision medicine, allowing patients to receive more effective treatments from the onset of symptoms. Since our study focused on TNF‐alpha antagonists, comparing the efficacy of this treatment modality alone versus combination therapies may help elucidate the mechanisms of action of multiple immunosuppressive drugs in sarcoidosis. Future studies should investigate infliximab and explore the effectiveness of other medications, such as adalimumab and etanercept.

Additionally, it is important to consider the correlation between the duration of TNF‐alpha antagonist usage (not limited to infliximab alone) and the relapse rate, as mentioned previously. A more extensive cohort study is needed to establish the success rate of adding infliximab for patients experiencing complications due to corticosteroid treatment and dosage tapering. This approach would provide valuable insights for subsequent monitoring and decision‐making in clinical practice.

Infection is a significant concern among patients receiving infliximab, an anticipated adverse event given the mechanism of TNF‐alpha inhibition. Our research did not observe adverse effects related to antibodies against infliximab, a phenomenon noted in some TNF‐alpha antagonist therapies due to its chimeric monoclonal antibody properties.^25^ However, it is essential to note that this does not entirely rule out such possibilities. Consequently, patients should receive essential care and adequate monitoring after receiving infliximab to minimize adverse outcomes as much as possible.

Based on our findings, infliximab may be considered as an alternative or adjunctive therapy in patients with refractory neurosarcoidosis due to its remarkable outcome in clinical improvement. Most of relapse cases were related to dose reduction or discontinuation of infliximab. Additionally, adverse events were not contributed to numbers of treatment discontinuation.

This systematic review and meta‐analysis had several limitations. First, the included studies had variations in the concurrent treatments administered and the severity of each patient's condition. This variation is inherent to the backgrounds of patients who typically receive TNF‐alpha antagonists after corticosteroids and antimetabolites have shown inadequate results. However, this variability could make it challenging to interpret infliximab's outcomes. At the time of this study, no randomized controlled trial had been conducted to directly compare isolated infliximab treatment with concurrent treatments. This situation further emphasizes the need for future research to address this gap.

Second, the usage of infliximab biosimilars was also considered in this study and may introduce a confounding factor. Some studies have suggested no significant difference in efficacy between the original infliximab and its biosimilars.^26^ However, switching from the original infliximab to a biosimilar may result in disease relapse, especially considering concerns about blood–brain barrier permeability.^27^

## Conclusions

This systematic review and meta‐analysis demonstrated a significant improvement in patients with refractory neurosarcoidosis treated with infliximab. Using infliximab reduced the relapse rate and allowed for corticosteroid dose tapering, thereby minimizing the adverse effects of prolonged corticosteroid use. However, infection remains the primary adverse event associated with infliximab treatment.

## Author Contributions

S.C., P.S, N.A. and J.J. contributed with conception and design, acquisition of data, analysis and interpretation of data, statistical analysis, and drafting and revision of the manuscript. W.O. and T.R. contributed with analysis and interpretation of data, and statistical analysis. W.N. contributed with conception and design, acquisition of data, analysis and interpretation of data, statistical analysis, drafting and revision of the manuscript, and study supervision.

## Conflict of Interest

The authors declare that there is no conflict of interest.

## Supporting information


Figure S1.
