# Supplementary material for: Infliximab in neurosarcoidosis: a systematic review and meta‐analysis
Source: Ann Clin Transl Neurol. 2023 Dec 12;11(2):466–76. doi: 10.1002/acn3.51968 (PMC10863903; doi:10.1002/acn3.51968)

**Supplement data**

- **Search term**
- **eTable 1.** The quality assessment of enrolled seven studies using the modified Newcastle-Ottawa scale.
- **Figure S1 and S2.** Funnel plot for publication bias analysis

**Search term**

The following search terms were used to search for papers in the scientific publication database.

#1 Neurological

#2 Sarcoidosis

#3 Neurosarcoidosis

#4 Infliximab

#5 Etanercept

#6 Adalimumab

#7 Rituximab

#8 ‘Tumor necrosis factor-alpha’

#9 1 AND 2

#10 2 OR 3

#11 4 OR 5 OR 6 OR 7 OR 8

#12 10 AND 11

**eTable 1.** The quality assessment of enrolled seven studies using the modified Newcastle-Ottawa scale.

| **Study** | **Selection** | **Comparability** | **Outcome** |
| --- | --- | --- | --- |
| Chakales P. A., et al (2022) | 🟊🟊🟊🟊 |  | 🟊🟊🟊 |
| Hilezian F., et al (2021) | 🟊🟊🟊 |  | 🟊🟊🟊 |
| Fritz D., et al (2020) | 🟊🟊🟊 |  | 🟊🟊🟊 |
| Lord J., et al (2020) | 🟊🟊🟊🟊 |  | 🟊🟊🟊 |
| Riller Q., et al (2019) | 🟊🟊🟊 |  | 🟊🟊🟊 |
| Cohen Aubart F., et al (2017) | 🟊🟊🟊 |  | 🟊🟊🟊 |
| Gelfand J. M., et al (2017) | 🟊🟊🟊 |  | 🟊🟊🟊 |

**Figure S1.** Funnel plot for publication bias analysis, including seven studies that were enrolled in the analysis of clinical outcomes.


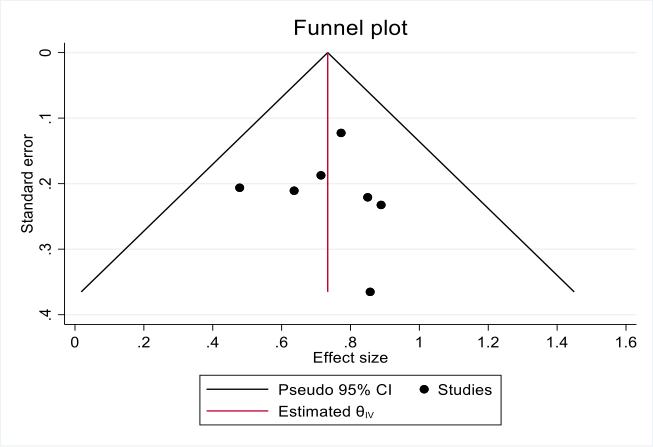


**Figure S2.** Funnel plot for publication bias analysis including four studies that were enrolled in the analyzing adverse events.


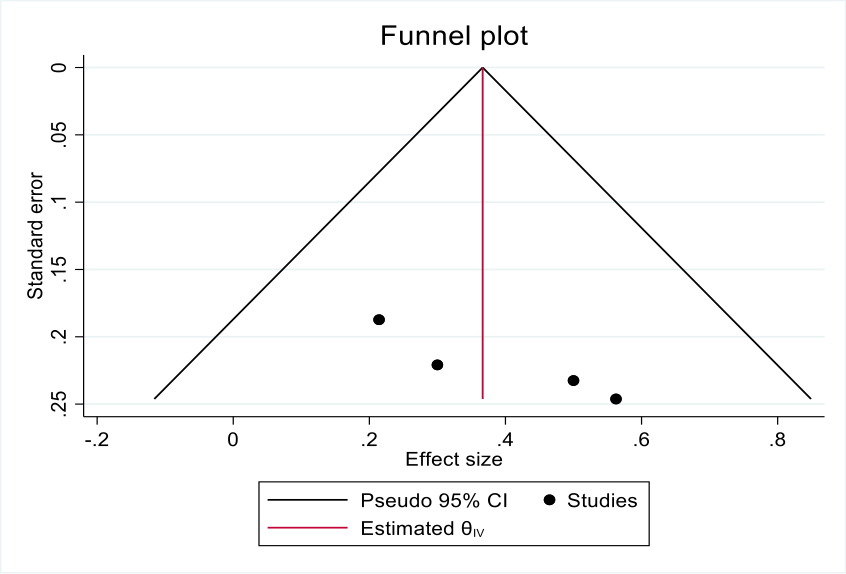

Supplement: Supplementary file 1 — Figure S1. [file ACN3-11-466-s001.docx]
